# REHABILITATION AND CARE AFTER HIP FRACTURE: A COST-UTILITY ANALYSIS OF STEPPED-WEDGE CLUSTER RANDOMIZED TRIAL

**DOI:** 10.2340/jrm.v56.40897

**Published:** 2024-11-21

**Authors:** Jonas A. IPSEN, Jan Abel OLSEN, Bjarke VIBERG, Lars T. PEDERSEN, Inge H. BRUUN, Eva DRABORG

**Affiliations:** 1 Department of Physical Therapy and Occupational Therapy, Lillebaelt Hospital, University Hospital of Southern Denmark, Kolding, Denmark; 2 Department of Regional Health Research, University of Southern Denmark, Odense, Denmark; 3 Department of Orthopaedic Surgery and Traumatology, Lillebaelt Hospital, University Hospital of Southern Denmark, Kolding, Denmark; 4 Department of Community Medicine, UiT The Arctic University of Norway, Tromsø, Norway; 5 Department of Orthopaedic Surgery and Traumatology, Odense University Hospital, Odense, Denmark; 6 Department of Clinical Research, University of Southern Denmark, Odense, Denmark; 7 Department of Health Education, University College South Denmark, Esbjerg, Denmark; 8 Danish Centre for Health Economics, Department of Public Health, University of Southern Denmark, Odense, Denmark

**Keywords:** hip fracture, QALY, community-dwelling patient, trial, cost, rehabilitation and informal care

## Abstract

**Objective:**

To estimate the effectiveness and costs of Rehabilitation for Life (RFL) compared with usual rehabilitation and care after hip fracture to determine which course offered the most value for money.

**Design:**

Cost-utility analysis.

**Patient:**

Community-dwelling patients aged 65+ after hip fracture.

**Method:**

123 intervention and 122 control patients were included. Data was collected at 5 points from discharge to 1-year follow-up. Cost analysis included expenses to hospital, general practice, specialist services, medications, rehabilitation, home and informal care, transport, and waiting times. The primary outcome was the incremental cost per quality-adjusted life year (QALY).

**Results:**

The intervention group experienced a statistically significant mean QALY gain of 0.02 compared with the control group. The intervention was more costly by €4,224, resulting in an incremental cost of €159,990 per QALY gained. Two municipalities had several patients in respite care, yielding an imbalance. A subanalysis excluding these patients demonstrated QALY gain at 0.03 and the cost difference of €2,586 was not statistically significant.

**Conclusion:**

The intervention demonstrated a slight improvement in effectiveness over the control but was costly. For patients not requiring respite care, the intervention effect was slightly higher, and the cost differences statistically insignificant. In total 91% received informal care and the economic contribution of informal care exceeded the municipal home care services.

## LAY ABSTRACT

Hip fractures are common, devastating, and costly to rehabilitate. The effectiveness of rehabilitation varies due to demand for comprehensive care from hospitals, municipalities, and family members. In this study, we evaluated 2 rehabilitation programmes to see which offered the best value for money. We monitored 123 patients in the intervention group and 122 in the control group over 1 year, assessing their quality of life at 5 different points. We calculated the 1-year difference in quality of life and included costs related to healthcare services, rehabilitation, home care, transportation, and support from relatives. While the intervention did slightly improve quality of life, it came with a high price tag. However, for more specific patient subgroups the intervention was better and less costly. Hence focusing on subgroups might be an economically viable next step. Additionally, the burden on relatives was high, demonstrating the extensive reliance on family for ongoing support.

Hip fractures are common, costly, and detrimental to older patients’ daily living and quality of life (QOL) (1, 2). Substantial resources are assigned to treatment, rehabilitation, and care to facilitate recovery (1–3). Nevertheless, only 40–60% of patients return to their pre-fracture mobility even 1 or 2 years after discharge (4). Rehabilitation and care are key interventions to facilitate recovery and resumption of independence. However, the effectiveness and cost of rehabilitation services and care varies on how much, when, and how it is delivered.

Globally, hip fracture cost estimates vary significantly, and to our knowledge none include all relevant costs from a societal perspective. For instance, informal caregiving is prevalent after hip fractures and valued at 2–4% of the gross domestic product (GPD) in Sweden and the Netherlands (5–9). Transportation to and from rehabilitation is free for patients who cannot transport themselves in Scandinavia (10–12). Additionally, rehabilitation services can be delivered individually or team-based. In team-based sessions, 1 physiotherapist supervises more patients simultaneously, which needs to be accounted for in the valuation. Hence the cost estimates associated with rehabilitation after hip fracture are likely imprecise.

Given the expected demographic developments in the population, the total costs of hip fractures will only increase in the future (13). At the same time, the influx of new and expensive treatments also puts pressure on the limited resources. Hence, prioritization is inevitable. However, information on costs and effects is imperative to prioritize resources efficiently. Two cost-utility analyses have evaluated the cost and effect of exercise interventions targeting older community-dwelling patients after hip fractures. Neither was cost-effective compared with usual care (3).

In 2020, a cluster-randomized stepped-wedge clinical trial, Rehabilitation for Life (RFL), was initiated. RFL assessed the effect of early resistance exercises and detection of critical illness and complications in an empowerment-orientated praxis. Compared with usual rehabilitation and care, RFL entailed more rehabilitation sessions, supervised team-based resistance exercises, and systematic follow-up of potential medical complications after discharge from municipal nurses (14). However, whether RFL offers better, worse, or similar patient outcomes is unknown, and that also applies to the associated costs, including, among others, informal care and transportation costs. This cost-utility analysis aimed to estimate the effectiveness and costs of RFL compared with usual rehabilitation and care after hip fracture to determine which course offered the best value for money.

## METHOD

### Health economic analysis plan

This study was a trial-based, cost-utility analysis. Reporting followed the updated Consolidated Health Economic Evaluation Reporting Standards statement (CHEERS) (15). A Health Economic Statistical Analysis Plan (SAP) was developed and uploaded to PURE University of Southern Denmark on 15 April 2024 before the measurement of costs was completed (16).

### Population

Inclusion criteria were community-dwelling, cognitively non-impaired patients aged 65 years or older who sustained hip fractures and consented to participate in the cost-utility analysis. Exclusion criteria were inability to speak or understand Danish, discharge from hospital to permanent residence in nursing homes, communication impairments, such as progressed dementia and aphasia, other disabling diseases making them unable to participate in rehabilitation, or short life expectancy.

### Setting and location

The Danish healthcare system is divided into 2 self-governing sectors. Regions cover hospitals, general practice, specialists, and prescription drugs, while municipalities cover rehabilitation and care outside hospitals, including home nursing services. Hospitals and municipalities are divided into catchment areas, each with 1 hospital and several municipalities. The healthcare system is a universal single-payer system, and rehabilitation and care are free of charge (17). One hospital and the 6 municipalities within the catchment area participated in this study. The catchment area serves a mixed urban and rural population. The responsibility for providing rehabilitation and care depends on the patient’s location (in hospital or at home) (17, 18).

### Comparator and intervention

*Usual rehabilitation and care.* All hip fracture patients receive surgery, mobilization, and care during their hospital stay. After discharge, a municipal rehabilitation programme is initiated. It usually consists of supervised exercise in the patient’s private home or at a rehabilitation center, encompassing 1 or 2 weekly sessions of 30 to 60 min each for 6–8 weeks (28). Municipal nursing is offered according to the patient’s needs.

*Intervention.* The RFL intervention was delivered in addition to usual rehabilitation and care and entailed continuous rehabilitation and care delivered in an empowerment-orientated praxis. The patients received 5 supervised resistance exercise sessions by municipal-employed physiotherapists during the first 2 weeks after discharge. The third of these sessions entailed a virtual meeting between the patient, 1 hospital physiotherapist, and 1 municipality physiotherapist. From week 3 to week 12, the patients received 20 resistance exercise sessions supervised by a physiotherapist from the municipality. Municipality-employed nurses conducted a home visit on day 3 after discharge. They assessed the patient’s health, including infection testing and, if needed, they could confer with medical doctors at the hospital. The empowerment-orientated praxis was intended to enable patients to gain control over their rehabilitation and care. It consisted of 3 initiatives: (*i*) medical information and knowledge were provided to the patients using a digital application (Mit Sygehus; https://regionsyddanmark.dk/patienter-og-parorende/hjaelp-til-patienter-og-parorende/mit-sygehus); (*ii*) the health professionals participated in a workshop where they were instructed on how to facilitate the empowerment of the patients; (*iii*) the patients received physical reminders through a trolley, a mug, weight cuffs, a printed exercise diary, and exercise programmes. A study protocol has been published for additional information on RFL and comparator (14).

### Perspective

The national retirement age is 67, and this study included only patients aged 65+, so a limited societal perspective, excluding production gains or losses, was used.

### Time horizon

The follow-up period was 1 year. Incremental costs and utility were assumed to be well established after 6 months, as most improvements after hip fracture occur within the first 6 months after discharge (4).

Due to the duration of the follow-up of 1 year, discounting was not applied.

### Selection of outcomes

*Primary outcome*. The primary outcome was the incremental cost per quality-adjusted life year (QALY). QALYs combine time lived and Health-Related Quality of Life (HRQoL), including items covering physical function and mental function, into a single index number where “1” corresponds to perfect health and “0” corresponds to being dead. HRQoL was measured using the EuroQol 5-dimension 5-level questionnaire (EQ-5D-5L) as a standardized questionnaire used to assess HRQoL (19). It comprised 5 dimensions: mobility, self-care, usual activities, pain, and anxiety/depression, each described using 5 severity levels (19). The patient’s HRQoL was assigned utility weights from the Danish EQ-5D-5L reference set (i.e., health states are assigned values on a scale between –0.759 and 1.000) (20). The outcome was reported as the total difference in QALYs as the area under the curve, from which the incremental cost per QALY gain was estimated.

*Secondary outcomes.* Demographic characteristics were age, sex, body mass index (BMI), living arrangement (i.e., living alone or cohabiting), and health status using the American Society of Anesthesiologists classification system (ASA) (31). The ASA score ranged from 1 to 6 and was dichotomized into a low-risk group (ASA 1–2) and a high-risk group (ASA ≥ 3) (21).

Mobility was measured using the clinician-applied 0–9 New Mobility Score (NMS) to assess the patient’s gait function indoors, outdoors, and during shopping. This score was measured on discharge, at 8 weeks, 12 weeks, 6 months, and 1 year after discharge (22).

Activities of daily living (ADL) were measured using Barthel-20 to assess a patient’s need for assistance (23). Barthel-20 measures the patient’s self-perceived ability to perform basic ADLs on a scale from 0 to 20 on discharge and 8 weeks, 12 weeks, 6 months, and 1 year after discharge.

*Resource consumption and valuation.* Hospital resource consumption included all in-hospital and outpatient contacts and services from admission to 6-month follow-up. Contacts and reimbursements were collected from the hospital’s administrative systems.

Municipal resource consumption was the extent of rehabilitation, nursing services, and homecare delivered by the municipalities from admission to 6-month follow-up. Rehabilitation was delivered individually or team-based (approximately 4 patients to 1 physiotherapist). We could not determine whether the municipalities delivered 1-to-1 or team-based rehabilitation using municipal registries. Hence, every 2 weeks during the first 3 months after discharge, the patients were contacted and asked how many rehabilitation sessions they had participated in and whether these sessions were 1-to-1 or team-based. The percentage of the total amount of rehabilitation sessions delivered as 1-to-1 was calculated for each group (1-to-1 session: control 68.0%, intervention 34.0%).

Respite stay resource consumption constituted temporary admissions to a municipal rehabilitation unit or nursing homes. These were offered if the patients were too frail to be discharged directly to their homes. The number of days in a respite stay was collected from the municipality’s administrative systems. The valuation reflected the municipality’s mean daily fee for 1 patient, including rehabilitation, care, nursing, and overhead charges for operating the unit and rehabilitating the patient. Municipalities provided the value.

Transportation resource consumption was estimated as 1 of 2 modes of transportation, either if rehabilitation was delivered in the patient’s home (physiotherapist travelled to the patient’s home) or in a municipal rehabilitation centre (patients travelled to the rehabilitation centre). We could not obtain information on transportation to and from rehabilitation from municipality registers. Thus, every second week during the first 12 weeks after discharge, the patients were contacted and asked how many rehabilitation sessions they had participated in at home or in a rehabilitation centre. The municipalities provided the value.

General practice resource consumption was the number of contacts with general practitioners and other private health professionals.

Other healthcare professionals’ resource consumption was the number of contacts with general practitioners and other private health professionals.

Prescription drug resource consumption was the number of cashed prescriptions.

Informal care (IC) resource consumption was obtained from patients who recorded in diaries the number of hours of informal care received from relatives. These were collected every 2 weeks for the first 12 weeks after discharge. The patients were instructed to record only the need for IC generated by the hip fracture and how long they received IC. Patients who did not fill in the diary were asked to estimate the hours of IC the previous week and to include both weeks; the estimate was multiplied by 2.

Waiting time resource consumption was obtained from patients. Transportation to and from rehabilitation sessions was delivered free of charge to the patients by the municipalities (by taxi). The same taxi picked up several patients, and to allow for flexibility in the planning the patients had to be ready to leave up to 1 hour before the scheduled time of arrival of the taxi. Patients were contacted every second week, for the first 12 weeks after discharge. They were asked how many rehabilitation sessions they received, where they were delivered (at home or in a rehabilitation centre), how they got to the rehabilitation centre (by taxi or travelling by themselves), and how much time they spent waiting and spending in transportation to the rehabilitation centre (Table I).

**Table I:** Resource consumption, unit, valuation and valuation source

Resource	Unit	Valuation	Valuation source
In-hospital	Days	Reimbursement per the diagnosis-related group (DRG)	Diagnosis-related group (DRG) (36)
Outpatient	Hours	Reimbursement per the diagnosis-related group	Diagnosis-related group (DRG) (36)
Rehabilitation	Hours	€46.2 for 1-to-1 sessions. €11.5 for team-based sessions	Hourly salary for a municipal physiotherapist + 40% to account for administration time (37)
Homecare	Hours	€47.0 per hour	Hourly salary for a municipal homecare assistant + 40% to account for administration time (37)
Community nursing	Hours	€54 per hour	Hourly salary for a municipal nurse + 40% to account for administration time (37)
Respite stay	Days	€327.7 per day	Municipalities mean cost per patients per day
Transport	Trips	€37 per round trip	Municipality’s mean cost per patient per round trip
General practice	Contacts	Fee per contact	National Health Service Register (38)
Other health practices	Contacts	Fee per contact	National Health Service Register (38)
Prescription medication	Prescriptions cashed	Fee per cashed prescription	National Register of Pharmaceutical Sales (39)
Informal care	Hours	€37.1 per hour	Standardized hourly earnings (37)
Waiting time	Hours	€37.1 per hour	Standardized hourly earnings (37)

### Data collection

A physiotherapist from the RFL trial contacted the patients 5 times during the 1-year follow-up period: on discharge, at 8 weeks after surgery, 12 weeks after surgery, 6 months after surgery, and 1 year after surgery. Measurement on discharge was carried out at the hospital, and the remaining 4 follow-ups were carried out during in-home visits and phone calls 1 year after discharge.

After a hip fracture, patients are in crisis, which affects their memory (35, 36). This, combined with the time between follow-ups, made it unlikely patients could recall detailed information. Hence, the cost of transportation, informal care, and waiting time were collected during the same bi-weekly phone interviews. Non-responders were contacted twice on 2 separate days before a missing data point was accepted (i.e., 4 telephone calls were performed) to mitigate missing data due to non-response to the phone call.

### Currency, price date, and conversion

Costs were collected in Danish Kroner (DKK), converted, and reported in euros (€) using the average 2023 conversion rate of €1 to DKK7.46 (24).

### Statistical analysis

We assessed the baseline characteristics of the population. For continuous variables, differences were assessed using Wilcoxon’s rank-sum test as variables did not follow a normal distribution. Reporting was in the median and interquartile range (IQR). Categorical variables were assessed using Pearson’s χ^2^ test, and reporting was in numbers and percentages. The cost was estimated as total costs between groups from surgery to 6-month follow-up and presented as aggregated and disaggregated in duration (e.g., hours or days) and monetary value. As we had several measurements on the same patients, an adjusted linear mixed regression model was used to estimate the change in utility between groups. The fixed effect parameter included time and group allocation (time#group), the random effect parameter included each individual as a cluster, and an interaction between time and group allocation was specified in the model.

*Yij* = *β*0 + *β*1 × *Timeij* + *β*2 × *Groupij* + *β*3 × (*Time* × *Group*)*ij* + *β*4 … + *ui* + ∈ *ij*

Y_*ij*_ was the utility score of the EQ-5D-5L for the *i*th individual and the *j*th timepoint. Hence, Y*ij* was the sum or fixed effect of time (β1) multiplied by the fixed effect of group (β2) plus the fixed effect of the interaction between time and group (β3) plus the fixed effect of each covariate (*β*4…) plus time at the *j*th timepoint (Time*ij*) + the group membership for the *i*th patient at the *j*th timepoint (Group*ij*). This was multiplied by the interaction between time and group (*Time* × *Group*)*ij* and a random effect for the *i*th patient (u*i*) and the random error (∈ *ij*). Model fit was tested using the Akaikes Information Criterion (AIC). We adjusted the model for the covariates that differentiate from zero at a significance level of 0.05 in a Wald χ^2^ test (age, ASA, cohabiting, surgery, mobility, and length of stay in hospital). There were no interactions between groups, and the model assumption was fulfilled. The health state of each individual at each time point was predicted. Using the predicted health states and time spent in these the individual patients’ QALY gain was calculated. The mean difference in QALY gain and cost was used to estimate the incremental cost-effectiveness ratio (ICER). Uncertainty of the ICER was estimated using bootstrapping, where each observation was reproduced by 1,000 bootstraps (25). Results were visualized in a cost-effectiveness plane and compared with the commonly used willingness-to-pay threshold (€20,000) per QALY (26, 27). Mixed regression accounts for missing data points, and Danish registries’ high completeness meant no imputations were done on utility or cost (27–29). As a sensitivity analysis, the analysis was run using a healthcare sector perspective. Three patients died during follow-up. They were imputed with a utility score of zero. An analysis was also run with these individuals excluded. Of the 25 patients in respite care, 19 were in the intervention group and caused group imbalances. A sub-analysis excluding these patients was therefore conducted. The impact of each dimension of the EQ-5D-5L was explored by estimating a change in mean level scores between groups over time. The significance level for all statistical analyses was set to 95%. Statistical analyses were performed with StataCorp 2019 (Stata Statistical Software: Release 18; StataCorp LLC College Station, TX, USA).

## RESULTS

**Fig. 1:**
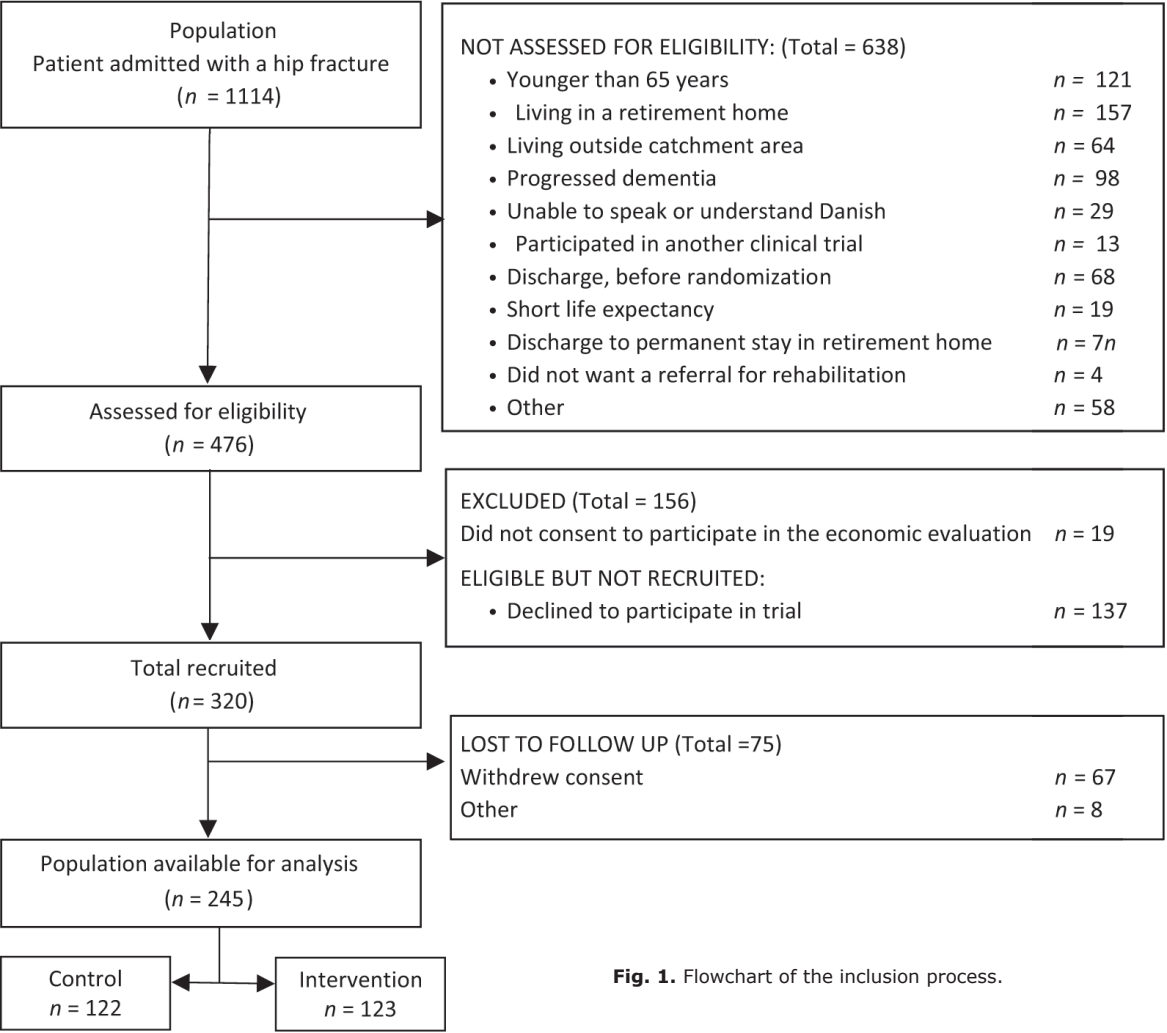
Flowchart of the inclusion process.

Patients were recruited from September 2020 to February 2023; 1,114 were screened, and 476 were eligible. Of those recruited, 67 withdrew their consent, and 8 were lost to follow-up. Thus, 122 were randomized to the control group and 123 to the intervention group. Their median age was 79 (IQR 74–84), and 164 were female (see Fig. 1). At baseline, the intervention and control groups were comparable (Table II).

**Table II:** Patient characteristics

Factor	Intervention: Rehabilitation For Life	Control: usual rehabilitation and care	Population
*n* (%)	123 (50.2)	122 (49.8)	245 (100.0)
Sex, *n* (%)			
Female	81 (65.9)	83 (68.0)	164 (66.9)
Male	42 (34.1)	39 (32.0)	81 (33.1)
Age, *n* (%)			
65–74 years	29 (23.6)	41 (33.6)	70 (28.6)
75–84 years	68 (55.3)	62 (50.8)	130 (53.1)
85+ years	26 (21.1)	19 (15.6)	45 (18.4)
ASA, *n* (%)			
Low	71 (57.7)	64 (52.5)	135 (55.1)
High	52 (42.3)	58 (47.5)	110 (44.9)
BMI, *n* (%)			
18.5–24.9	64 (52.0)	55 (45.1)	119 (48.6)
< 18.4	4 (3.3)	7 (5.7)	11 (4.5)
25–29.9	39 (31.7)	42 (34.4)	81 (33.1)
30+	16 (13.0)	18 (14.8)	34 (13.9)
Cohabiting, *n* (%)			
Living with a partner	65 (52.8)	58 (47.5)	123 (50.2)
Living alone	58 (47.2)	64 (52.5)	122 (49.8)
Surgery, *n* (%)			
Arthroplasty	41 (33.3)	42 (34.4)	83 (33.9)
Sliding hip screw	28 (22.8)	32 (26.2)	60 (24.5)
Intramedullary nail	54 (43.9)	48 (39.3)	102 (41.6)
Mobility			
Independent	96 (78.0)	86 (70.5)	182 (74.3)
Dependent on others	27 (22.0)	36 (29.5)	63 (25.7)

ASA: American Society of Anesthesiologists classification system.

### Utility

The intervention group had a statistically significant higher utility gain 6 months after the hip fracture (*p* < 0.03); however, after 1 year, there was no statistically significant difference between the intervention and control group (*p* > 0.76). No statistically significant difference was observed in mobility, ADL, or the EQ-VAS score 1 year after the hip fracture. The utility gain ranged from 0.00 in the crude ICER estimate to 0.03 in the adjusted ICER estimate, where patients discharged to a respite stay were excluded (Table III).

**Table III:** Clinical outcomes

Factor	Intervention: Rehabilitation for Life			*p*-value
	Median	IQR	IQR	
Discharge				
EQ-5D value	0.51	0.26 0.68	0.24 0.67	**0.02**
EQ VAS score	55	50 75	50 75	0.45
Mobility	2	1 3	1 4	**0.04**
ADL	14	10 17	12 17	0.06
8 weeks				
EQ-5D value	0.65	0.48 0.75	0.40 0.76	0.48
EQ VAS score	70	50 80	50 80	0.86
Mobility	6	4–7	4 7	0.17
ADL	19	18 20	17 20	0.29
12 weeks				
EQ-5D value	0.74	0.63 0.80	0.56 0.80	0.38
EQ VAS score	75	50 84	50 85	0.73
Mobility	6	5 9	4 8	**0.03**
ADL	19	18 20	17 20	0.35
6 months				
EQ-5D value	0.76	0.69 0.83	0.58 0.81	**0.03**
EQ VAS score	75	50 85	50 84	0.20
Mobility	7	6 9	5 9	0.09
ADL	20	19 20	18 20	**0.02**
1 year				
EQ-5D value	0.72	0.55 0.81	0.63 0.81	0.76
EQ VAS score	75	60 84	50 90	0.23
Mobility	7	6 9	6 9	0.18
ADL	20	18 20	18 20	0.27
QALY				
QALY crude			0.00 (–0.01; 0.00)	
QALY adjusted			0.02 (0.00; 0.05)	
QALY adjusted patients dying excluded			0.01 (–0.00; 0.04)	
QALY adjusted patients in respite stay excluded			0.03 (0.01; 0.06)	

EQ-5D value: the EuroQol 5-item 5-level questionnaire (EQ-5D-5L), with values based on the Danish value set.

EQ VAS: the EuroQol Visual Analogue Scale VAS (0–100).

QALY: Quality adjusted life year.

Adjustments: age, ASA, cohabiting, surgery, mobility, and length of stay in hospital.

### Resource consumption

The median total cost of the intervention was €5,581 (*p* < 0.00) higher in the intervention group. From a narrower healthcare sector perspective, the difference was €4,294 (*p* < 0.01) higher for the intervention group. The major cost drivers differentiating the intervention from the control were respite stay (difference €4,751), rehabilitation (difference €505), general practitioner (difference €336), waiting time (difference €992), and informal care (€–482) (Table IV).

**Table IV:** Cost

Factor	Intervention: Rehabilitation For Life (€)	Control: usual rehabilitation and care (€)	Differences in costs (€)
Median (IQR)	123 (50)	122 (50)	245 (100)
In-hospital	9,253 (8,177; 13,535)	8,983 (8,497; 10,541)	270
Outpatient	442.5 (154; 1,724)	214.8 (84; 829)	227
Rehabilitation	1,193 (900; 1,697)	688 (323; 1,164)	505
Homecare	1,163 (188; 5,123)	1,165 (60; 5,755)	–2
Community nursing	364.8 (155.7; 978.3)	594 (119; 1,382)	–229
Respite stay	13,271 (8,520; 20,727)	8,520 (4,423; 14,746)	4,751
Transport	384 (192; 552)	48 (24;144)	133
General practitioner	476 (186; 760)	343 (148; 693)	336
Other health practitioners	145 (29; 340)	150 (0; 288)	–5
Prescription drugs	55 (25; 110)	54 (29; 98)	0
Informal care	1,075 (519; 2,476)	1,558 (630; 2,893)	–482
Waiting time	1,137 (612; 2,285)	145 (49; 728)	992
Total cost limited societal perspective	21,938 (15,477; 33,957)	16,357 (12,345 ; 29,259)	5,581
Total cost healthcare sector perspective	17,994 (12,037; 29,164)	13,699 (10,962; 25,461)	4,294

### ICER estimate

**Fig. 2:**
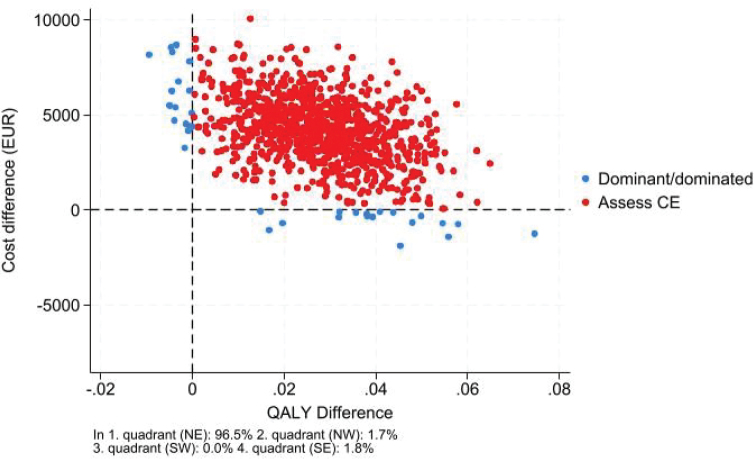
ICER plane visualizing the 1,000 bootstrapped observations reproduced to the societal perspective.

**Fig. 3:**
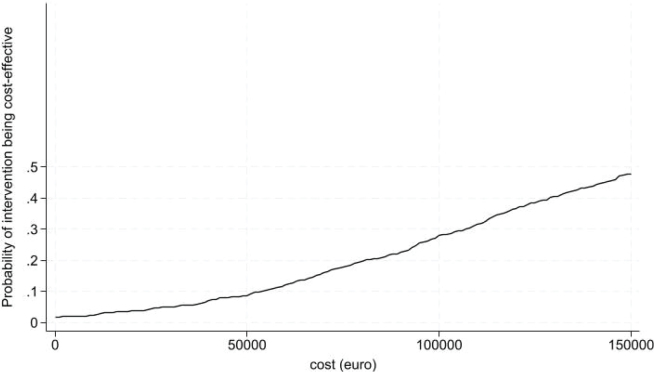
CEAC curve: visualizing the probability of the intervention being cost-effective to the societal perspective.

There was a small, but statistically significant, difference in QALY gain of 0.02 (95% CI 0.00; 0.05) in favour of the intervention. The cost difference was €4,224 (95% CI 7€22; €7,727) favouring the control. The incremental cost per QALY gained was €159,990. Of the bootstrapped observations, 96% were in the northeast corner of the ICER plane, indicating that patients receiving the intervention had better outcomes at a higher cost (Fig. 2). The probability of the intervention being cost-effective was 7% (Fig. 3). There was no significant variation in secondary outcomes or between the intervention and control on the mean level scores of the dimensions of the EQ-5D.

Utilizing a healthcare perspective (i.e., informal caregiving and waiting time excluded) reduced the cost difference but did not change the ICER. Excluding the patient’s discharge to a respite stay increased the QALY gain to 0.03 (95% CI 0.01; 0.06) and decreased the incremental costs to €2,586 (95% CI –€674, €5,847) and the incremental cost per QALY was €67,531 and statistically insignificant (Figs S1 and S2).

In total, 91% of the population received informal care from relatives, which accounted for 7% of the total median cost and exceeded the cost associated with formal care.

## DISCUSSION

### Summary of findings

The Rehabilitation for Life (RFL) study demonstrated a minor, yet statistically significant, improvement in quality adjusted life years (QALY), albeit with significantly higher costs. Removing indirect costs reduced the overall expense, but RFL remained costlier. Excluding patients in respite care slightly increased QALY gains, drastically lowered incremental costs to a statistically insignificant level, and for 7% the intervention was better and less costly. Hence the intervention should not be offered to the entire sub-population indiscriminately, but for some subgroups – for instance patients discharged to their own homes – the intervention is potentially viable.

A notable finding was the substantial role of informal care provided by relatives, reflecting a broader trend where responsibilities have increasingly shifted from hospitals to municipalities, leading to faster patient discharges compared with a decade ago. This shift has seemingly extended to informal caregivers as well, who now bear more responsibilities (9, 30). This hypothesis is supported by findings by Statistics Denmark, who report that elders with partners receive formal care later and when they receive it they receive more than patients without partners (31).

### Interpretation

Previous cost-utility analyses of rehabilitation interventions following hip fracture include the study by Milte et al. (32), which compared exercise and nutritional intervention with usual care. Their findings showed a statistically insignificant QALY gain of 0.02 and a mean cost difference of 567 AUD (€≈347) (32). In contrast, the QALY gain identified in our study was similar but reached statistical significance. This discrepancy could stem from differences in how QALY gain was calculated: In our study, QOL was assessed 5 times from discharge over the course of a year, and we utilized a linear mixed model (LMM) (31). The LLM approach allowed us to include both fixed and random effects, capturing variations within patients over time and thus reducing uncertainty around our estimated QALY gain (32). Another relevant study by Taraldsen et al. (33) examined a late-phase exercise intervention compared with usual care and found no difference in QALY or costs. When compared with the analyses by Milte et al. (33), our Rehabilitation For Life intervention appears costlier. Our measurement of costs was more comprehensive, encompassing both direct and indirect costs, and RFL intervention included video conferences and hotlines linking physiotherapists and nurses across hospitals and municipalities. It also extended services to patients discharged to respite care, who require more intensive observation and rehabilitation due to their frailty. Hence this cost difference was somewhat expected. Extending the intervention for patients in respite stay did markedly increase the intervention cost, as these patients were not evenly distributed between intervention and control. This was due to organizational differences between municipalities where municipalities in clusters 1, 2, and 3 had very different policies in access to and duration of stay in respite.

### Strength and limitations

Our study utilized the 5-level EQ-5D-5L, which is more responsive to changes than the 3-level version (33–35), and we repeatedly measured QOL during the 1-year follow-up. We conducted an extensive measurement of costs, collecting all municipal costs directly from municipal administrative systems, an approach confirmed by data managers to include some homecare services not recorded in national registries. Transportation and informal care costs are undeniably relevant as they affect nearly all patients in this study. Our thorough and broad measurement of costs is a clear strength of this study. As the trial was conducted over 3 years and we had several measurements on each patient, the mixed regression analysis reduced the uncertainty of patients’ utility gain.

A significant limitation is that transportation, waiting time, and informal care costs were measured for only 3 months, while other costs were measured for 6 months, potentially leading to an underestimation of incremental costs. Moreover, we assumed costs to be incremental 6 months post-hip fracture based on findings by Dyer et al. (4), which suggested that major functional improvements occur within the first 6 months post-surgery. The control and intervention groups showed diminishing utility scores from the 6-month to the 1-year follow-up, which might indicate rising home care costs, thus possibly underestimating incremental costs. Despite this, the larger decline in quality of life in the intervention group suggests that extending the follow-up to 1 year would unlikely alter the study’s conclusions. In total 19 patients were excluded as they did not consent to participate in the cost-utility analysis. These 19 patients all belonged to the control and they had an extreme effect on mortality rates in favour of the control, when in fact there was no difference in mortality rates between intervention and control. Hence, we saw no other option than to exclude patients who died before the 6-month follow-up in the intervention and control. It had no effect on the conclusion or findings as there was no difference in mortality between groups. However, it reduced the generalizability of findings to a sub-group of patients with a lower mortality risk.

### Conclusion

This study reveals that while the Rehabilitation for Life (RFL) intervention marginally enhances QALY, it also incurs significantly higher costs than usual rehabilitation and care. The RFL intervention showed slightly improved outcomes for patients discharged to their homes, potentially without additional costs. The findings are limited to a healthier subgroup. Moreover, the study indicates that most (91%) patients received familial support, with the economic contribution of this informal care exceeding that provided by municipal homecare services. This shift suggests a crucial point for consideration by both policymakers and researchers: the ongoing reallocation of caregiving responsibilities from hospitals to families, prompted by changes in the roles between hospitals and municipalities.

## Supplementary Material

REHABILITATION AND CARE AFTER HIP FRACTURE: A COST-UTILITY ANALYSIS OF STEPPED-WEDGE CLUSTER RANDOMIZED TRIAL
