# Supplementary material for: REHABILITATION AND CARE AFTER HIP FRACTURE: A COST-UTILITY ANALYSIS OF STEPPED-WEDGE CLUSTER RANDOMIZED TRIAL
Source: J Rehabil Med. 2024 Nov 21;56:40897. doi: 10.2340/jrm.v56.40897 (PMC11600605; doi:10.2340/jrm.v56.40897)

Supplementary material has been published as submitted. It has not been copyedited, or typeset by Journal of Rehabilitation Medicine

Fig. S1. ICER for Patients discharge to own home

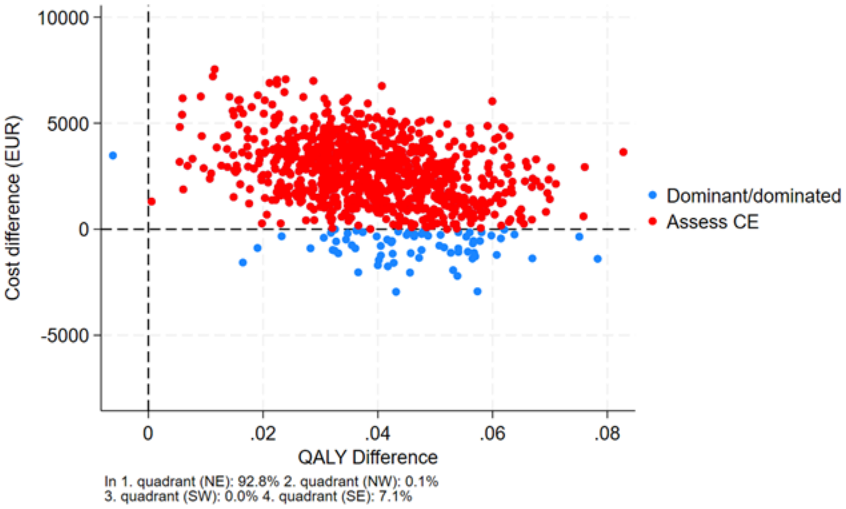

Fig S2. CEAC for patients discharged to their own home

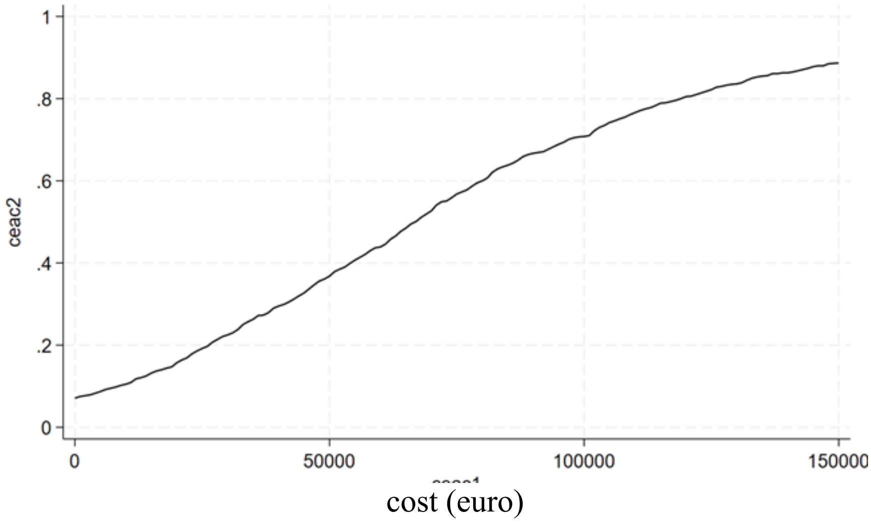

Supplement: REHABILITATION AND CARE AFTER HIP FRACTURE: A COST-UTILITY ANALYSIS OF STEPPED-WEDGE CLUSTER RANDOMIZED TRIAL [file JRM-56-40897-s1.pdf]
